# Impact of Preventive Intravenous Amiodarone on Reperfusion Ventricular Fibrillation in Patients With Left Ventricular Hypertrophy Undergoing Open-Heart Surgery: Randomized Controlled Clinical Trial

**DOI:** 10.2196/64586

**Published:** 2025-01-13

**Authors:** Chan-Juan Gong, Xiao-Kai Zhou, Zhen-Feng Zhang, Yin Fang

**Affiliations:** 1 Department of Anesthesiology and Perioperative Medicine, The First Affiliated Hospital of Nanjing Medical University, No. 300 Guangzhou Road, Nanjing, 210029, China, 86 2568303569

**Keywords:** amiodarone, left ventricular hypertrophy, reperfusion ventricular fibrillation, open-heart surgery, randomized controlled trial, RCT, clinical trial, ventricular fibrillation, vicious arrhythmia, aortic cross-clamp, surgery, effectiveness, defibrillation

## Abstract

**Background:**

Ventricular fibrillation (VF) is a vicious arrhythmia usually generated after removal of the aortic cross-clamp (ACC) in patients undergoing open-heart surgery, which could damage cardiomyocytes, especially in patients with left ventricular hypertrophy (LVH). Amiodarone has the prominent properties of converting VF and restoring sinus rhythm. However, few studies concentrated on the effect of amiodarone before ACC release on reducing VF in patients with LVH.

**Objective:**

The study was designed to explore the effectiveness of prophylactic intravenous amiodarone in reducing VF after the release of the ACC in patients with LVH.

**Methods:**

A total of 54 patients with LVH scheduled for open-heart surgery were enrolled and randomly divided (1:1) into 2 groups—group A (amiodarone group) and group P (placebo-controlled group). Thirty minutes before removal of the ACC, the trial drugs were administered intravenously. In group A, 150 mg of amiodarone was pumped in 15 minutes. In group P, the same volume of normal saline was pumped in 15 minutes. The primary outcome was the incidence of VF 10 minutes after removal of the ACC.

**Results:**

The incidence of VF was lower in group A than in group P (30% vs 70%, *P*=.003). The duration of VF, the number of defibrillations, and the defibrillation energy were also lower in group A than in group P (*P*<.001, *P*=.002, and *P*=.002, respectively). After the end of cardiopulmonary bypass, the heart rate and mean arterial pressure were lower in group A, and the mean pulmonary arterial pressure and the dose of vasoactive drugs were higher than those in group P (*P*<.001, *P*<.001, *P*=.04, and *P*=.02, respectively). However, there were no significant differences in the use of vasoactive-inotropic agents and hemodynamic status between the 2 groups before the end of surgery.

**Conclusions:**

In patients with LVH who undergo open-heart surgery, amiodarone can be safely used to reduce the incidence of VF, the duration of VF, the frequency of defibrillation, and the energy of defibrillation after ACC removal.

## Introduction

Ventricular fibrillation (VF) is commonly seen after release of the aortic cross-clamp (ACC) during open-heart surgery, which can result in reperfusion VF (rVF) when reperfusion of the myocardium begins. The incidence of rVF after the ACC release has been reported to range from 10% to 80% [1]. It has been proven in the animal model that persistent VF could cause more myocardial fibrosis [2]. The use of internal electrical defibrillation is a successful approach to terminate VF. However, multiple shocks and prolonged VF duration may exacerbate myocardial damage and diminish the patient’s prognosis [3]. Besides, repeated defibrillations would increase the incidence of postoperative bradycardia in cardiac surgeries [4]. Therefore, it is necessary to reduce the rVF after removal of the ACC during cardiac surgery.

In patients with left ventricular hypertrophy (LVH), the unique myocardial structure hinders the complete perfusion of fluid during cardiopulmonary bypass (CPB) surgery to reach the subendocardial cardiomyocytes. This limitation diminishes the efficacy of myocardial protection in open-heart surgery [5]. Following the release of the ACC, there is a notable rise in the occurrence of rVF, leading to an escalation in both the energy and frequency of cardiac shocks within the hypertrophic myocardium [6]. Thus, it is also of vital clinical significance to decrease the occurrence of rVF in patients with LVH.

Amiodarone, known for its classification as a potent Class III antiarrhythmic drug with diverse electrophysiological properties, has been extensively used in clinical practice for the treatment of atrial and ventricular arrhythmias [7,8]. Intravenous amiodarone has been confirmed to protect the myocardium from ischemia-reperfusion injury and prevent reperfusion-induced VF [9]. It also showed a good hemodynamic tolerance when administered intravenously [10]. Previous studies have demonstrated the efficacy of amiodarone in reducing the occurrence of VF after the removal of the ACC [11,12]. However, there is only 1 clinical trial investigating the role of amiodarone in reducing rVF in patients with LVH [13]. Therefore, we designed the study to investigate the effects of prophylactic intravenous amiodarone on rVF after the release of the ACC in patients with LVH undergoing open-heart surgery.

## Methods

### Settings and Patients

This was a prospective, double-blind, randomized controlled trial conducted in the First Affiliated Hospital of Nanjing Medical University, Nanjing, China. The patients undergoing open-heart surgery were screened by preoperative transthoracic echocardiography (TTE). When meeting the eligible criteria, the patients were recruited in the study. A total of 54 patients with LVH undergoing open-heart surgery were enrolled and participated in this study from August 2022 to November 2023. This manuscript adheres to all applicable CONSORT (Consolidated Standards of Reporting Trials) guidelines (Checklists 1 and 2). The protocol of this study has already been published [14]. The eligibility criteria are hence described.

The inclusion criteria included (1) patients undergoing open-heart surgery, (2) aged between 18 and 75 years, (3) diagnosed with LVH evaluated by TTE preoperatively, and (4) classified as American Society of Anesthesiology (ASA) Physical Status I-III. LVH was characterized by a left ventricular mass index>115 g/m^2^ for men and >95 g/m^2^ for women [15].

The exclusion criteria included preoperative administration of Class I or III antiarrhythmic medications; QT interval of 500 milliseconds or higher or corrected QT interval of 480 milliseconds or higher; left ventricular ejection fraction below 40%; and presence of thyroid dysfunction, electrolyte imbalances, liver or renal dysfunction, pulmonary interstitial fibrosis, or known allergy to amiodarone. Patients undergoing emergency surgery, redo surgery, or concomitant procedures, including coronary artery bypass grafting (CABG), were also excluded.

### Ethical Considerations

This study was approved by the Ethical Committee of the First Affiliated Hospital of Nanjing Medical University (2020-SR-175) and registered in the Chinese Clinical Trial Registry (ChiCTR2000035057). All participants provided verbal informed consent before being enrolled in the study. To secure and protect all participant information, all data were recorded by the specific ID number instead of the participant’s name throughout the study. Only select research team members could access these data. The process was monitored by the Ethical Committee of the First Affiliated Hospital of Nanjing Medical University.

### Randomization and Blinding

After anesthetic induction, the enrolled participants were assigned with a random permuted block method in a 1:1 ratio to 2 groups, namely, group A (treated with amiodarone) and group P (placebo control). An unblinded nurse anesthetist prepared the drugs according to the allocation information in the randomization envelope. The drugs were uniformly packaged, numbered, and administered by anesthesiologists. All involved parties, including the anesthesiologists, surgeons, and participants, were unaware of the specific treatment regimen, ensuring a blinded study design. However, nonblinded nurse anesthetists were not involved in evaluating treatment interventions, analyzing data, or interpreting results in the study.

### Treatment Regimen of Amiodarone

The trial drugs were administered 30 minutes before ACC release through the central venous line. In group A, 150 mg of amiodarone was diluted to 20 ml of normal saline and pumped in 15 minutes. In group P, 20 ml of normal saline was pumped in 15 minutes.

### Anesthesia Protocols

All patients received standard general anesthesia. In the operating room, patients were routinely monitored, including 5-lead electrocardiograph, peripheral blood oxygen saturation, invasive arterial blood pressure, bispectral index, and end-tidal CO_2_. Anesthesia induction was carried out as follows: 0.05 mg/kg midazolam, 0.3 mg/kg etomidate, 0.15 mg/kg cis-atracurium, and 4‐6 μg/kg fentanyl. Approximately 5 minutes after induction, patients were intubated and ventilated to maintain end-tidal CO_2_ at 35‐45 mm Hg. Then, ultrasound-guided central vein catheterization was performed through the right internal jugular or subclavian vein. The Swan-Gans catheter was floated to the pulmonary artery through the right internal jugular vein. The patients continued to receive anesthesia by inhalation of sevoflurane 1%‐2% and continuous pump of propofol 1‐2 mg/kg/h, dexmedetomidine 0.2‐0.7 μg/kg/min, and cis-atracurium 0.05‐0.1 mg/kg/h. The total dose of fentanyl was 30‐50 μg/kg. The bispectral index was maintained between 40 and 60. Inotropic agents, vasopressors, vasodilators, or fluid administration were applied to achieve the following hemodynamic status: a mean arterial pressure (MAP) of 50‐80 mm Hg, cardiac index (CI) ≥2.2 L/min/m^2^, and urine output ≥1.0 mL/kg/h.

### Surgical and CPB Procedures

The surgical procedures were performed by median sternotomy with heparinization and standard CPB. Anticoagulation was started with 400 U/kg of heparin and maintained according to an activated clotting time of >480 seconds. The CPB was initiated using a membrane oxygenator and the circuit was primed with 1500 ml of Ringer acetate solution and 200 ml of 50% human serum albumin. The patients were perfused with a nonpulsatile roller pump at a flow rate of 2.0‐2.4 L/min/m^2^ and with MAP of 50‐80 mm Hg. Moderate systemic hypothermia was maintained at 30‐32℃ during CPB. Myocardial protection was achieved by intermittent antegrade or retrograde infusions of histidine-tryptophane-ketoglutarate cardioplegia. After weaning from CPB, the reversal of heparin was achieved with protamine.

### Management of VF

Thirty seconds before ACC release, 1 mg/kg of esmolol was administered to all patients. When VF occurred after ACC release, internal electrical defibrillation with 20 J was performed in the first attempt. If not successful, the second defibrillation with 20 J was attempted after reusing 0.5‐1 mg/kg of esmolol. If VF persisted, the third 30 J defibrillation was performed after giving 1 mg/kg of lidocaine. If termination of VF was not achieved, the fourth defibrillation with 40 J was attempted after the administration of 50 mg of amiodarone administration. If VF still existed, the administration of 100 mg of amiodarone and defibrillation with 50 J was repeated until VF terminated.

### Outcome Measures

The primary outcome was the incidence of VF 10 minutes after ACC release. The secondary outcomes included the duration of VF, the number and total energy of defibrillations needed to terminate VF, and the use of other antiarrhythmic agents and epicardial temporary pacemakers after the release of the ACC. Furthermore, MAP, heart rate (HR), mean pulmonary arterial pressure (MPAP), central venous pressure (CVP), and CI were measured 15 minutes after anesthetic induction (T1), 15 minutes after weaning from CPB (T2), and 15 minutes before the end of surgery (T3). In addition, the vasoactive-inotropic score (VIS) and the total dose of vasoactive and inotropic agents (TDA) were also recorded at T2 and T3 (VIS = dopamine [μg/kg/min] + dobutamine [μg/kg/min] + 10 × milrinone [μg/kg/min] + 100 × epinephrine [μg/kg/min] + 100 × norepinephrine [μg/kg/min] + 1000 × vasopressin [U/kg/min]; TDA = dopamine [mg] + dobutamine [mg] + 10 × milrinone [mg] + 100 × epinephrine [mg] + 100 × norepinephrine [mg] + 1000 × vasopressin [U]).

### Sample Size Estimation

The sample size was calculated based on the primary outcome using PASS 15.0 (NCSS, LLC). According to published studies, the incidence of VF after ACC release ranged from 10% (2/20) to 80% (38/47) [1]. Based on our preliminary data, the incidence of VF 10 minutes after ACC release was 70% (7/10) in the placebo-controlled group, while the VF rate was 30% (3/10) in the amiodarone group. To reach statistical significance with a power of 80% and an α value of .05 (2-sided), 21 patients were needed in each group. Assuming a 20% dropout or missing data rate, we recruited 27 patients in each group for a total of 54 patients.

### Statistical Analysis

All data were analyzed using SPSS 22.0 (IBM Corp) and GraphPad Prism 8.0 (GraphPad Software Inc). All statistical tests were considered significant if *P*<.05. Only 2-sided tests were used. The normality assumption was assessed with the Kolmogorov-Smirnov test for all tests. Continuous variables were presented as mean (SD) or median (IQR), depending on the distribution. Categorical variables were presented as frequency (%). Continuous variables were compared using Student *t* tests if normally distributed or Mann-Whitney *U* tests if not normally distributed. The proportions were compared using Pearson chi-square or continuity correction tests. Data for repeated measures were compared using repeated measures ANOVA.

## Results

### Baseline Characteristics

**Figure 1.**
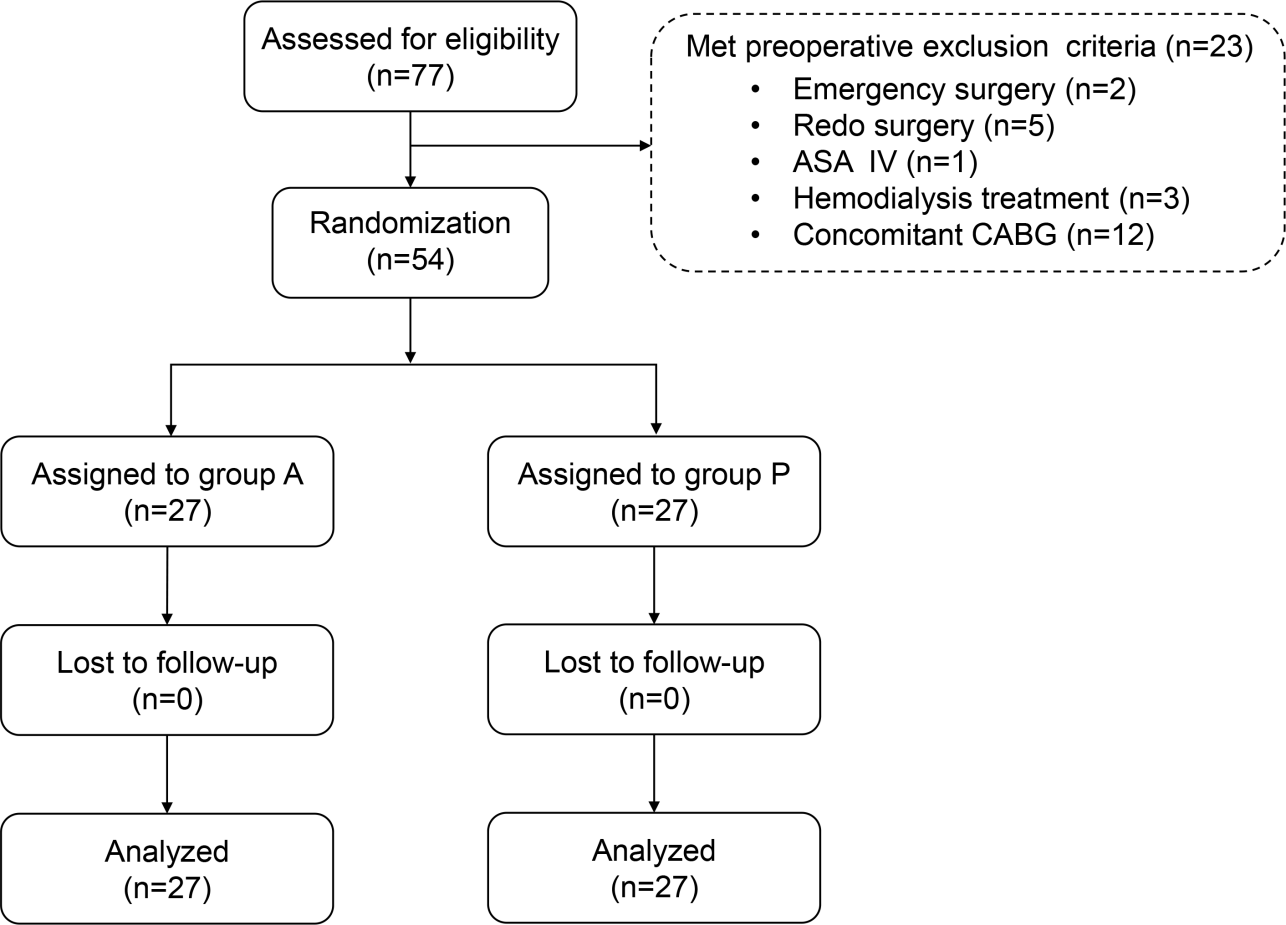
CONSORT (Consolidated Standards of Reporting Trials) flow diagram of patient recruitment, randomization, and withdrawal. A total of 27 patients in each group were eventually included in the final analysis. ASA: American Society of Anesthesiologists; CABG: coronary artery bypass grafting.

In total, 77 patients with LVH who underwent open-heart surgery were selected for this study. Among them, 54 patients (27 in group A and 27 in group P) completed this trial and 23 patients were excluded, which were shown in the CONSORT flow diagram (Figure 1). The demographic and perioperative data of the participants were comparable between groups (Table 1).

**Table 1.** Demographic characteristics and perioperative data.

Perioperative information			Group A^a^ (N=27)	Group P^b^ (N=27)	*P* value
**Preoperative**					
	Age (y), mean (SD)		64.44 (9.94)	65.59 (6.30)	.62
	Male, n		19	14	.16
	BMI (kg/m^2^), mean (SD)		22.74 (2.42)	23.50 (1.92)	.21
	Hypertension, n		10	6	.23
	Diabetes mellitus, n		4	7	.50
	Hyperlipidemia. n		3	2	.99
	Smoking, n		6	4	.73
	NYHA^c^ class, mean (SD)		2.63 (0.49)	2.41 (0.50)	.11
	LVMI^d^ (g/m^2^), mean (SD)		134.40 (11.74)	132.44 (15.05)	.60
	LVEF^e^ (%), mean (SD)		57.11 (3.94)	58.78 (5.51)	.21
	Calcium blockers, n		6	8	.54
	β-blockers, n		20	16	.19
	Diuretics, n		7	6	.75
	ACEIs^f^, ARBs^g^, n		10	11	.78
**Intraoperative**					
	**Procedures, n**				.20
		AVR^h^	18	23	
		Morrow	9	4	
	Surgical time (min), mean (SD)		358.44 (35.67)	375.04 (35.55)	.09
	CPB^i^ time (min), mean (SD)		154.89 (30.98)	168.70 (33.48)	.12
	ACC^j^ time (min), mean (SD)		135.33 (28.91)	143.81 (29.53)	.29
	Time for CPB weaning (min), mean (SD)		28.04 (10.81)	30.59 (8.69)	.42
	Blood loss (mL), mean (SD)		528.89 (213.87)	496.74 (184.61)	.56
	Urine output (mL), mean (SD)		1350.00 (527.36)	1417.04 (754.10)	.71
	Total fluid volume (mL), mean (SD)		3727.04 (423.72)	3562.59 (448.78)	.17
	Total cardioplegia (mL), mean (SD)		1385.19 (317.39)	1412.96 (374.06)	.77
	Abnormal electrolyte or acid-base metabolism, n		0	0	.99
**Postoperative, mean (SD)**					
	Ventilation time (h)		10.26 (4.69)	11.44 (4.93)	.37
	ICU^k^ stay (d)		4.96 (3.20)	5.93 (3.76)	.32
	Hospital stays (d)		11.85 (4.02)	13.74 (5.41)	.15

a Group A: amiodarone group.

b Group P: placebo control group.

c NYHA: New York Heart Association.

d LVMI: left ventricular mass index.

e LVEF: left ventricular ejection fraction.

f ACEI: angiotensin converting enzyme inhibitor.

g ARB: angiotensin receptor blocker.

h AVR: aortic valve replacement.

i CPB: cardiopulmonary bypass.

j ACC: aortic cross-clamp.

k ICU: intensive care unit.

### Intraoperative Outcomes

The incidence of VF after the release of the ACC was significantly lower in group A than in group P (8/27, 30% vs 19/27, 70%; *P*=.003) (Table 2). Furthermore, the number of internal shocks and the total energy of defibrillations were lower in group A than in group P (*P*=.002). Furthermore, a shorter duration of VF was also observed in group A (*P*<.001). Regarding the restoration of cardiac rhythm after ACC release, there were no significant differences in the recovery time of cardiac contraction or the proportion of epicardial pacing between the groups. However, the duration of epicardial pacemaker use in group A was higher than those in group P (*P*=.03). When dealing with VF after the release of the ACC, there were no significant differences in the consumption of antiarrhythmic drugs between the groups (Table 3). At the T2 time point, group A had a higher VIS than group P (*P*=.02). However, no differences in VIS were observed between the groups at the time of T3. During the whole intraoperative period, there were no significant differences in TDA between the groups.

**Table 2.** Incidence of ventricular fibrillation (VF) and restoration of cardiac rhythm after aortic cross-clamp release.

Changes of cardiac rhythm		Group A^a^ (N=27)	Group P^b^ (N=27)	*P* value
VF, n (%)		8 (30)	19 (70)	.003
**Defibrillations, n**				.002
	0	19	8	
	1	6	9	
	≥2	2	10	
Total energy (J), median (IQR)		0 (0‐20)	20 (0‐40)	.002
VF duration (min), median (IQR)		0 (0‐1)	1.5 (0‐3.5)	<.001
Time for cardiac contraction recovery (min), mean (SD)		2.63 (1.15)	2.61 (1.60)	.96
Epicardial pacing, n		14	8	.10
Duration of epicardial pacing (min), median (IQR)		10 (0‐41)	0 (0‐9)	.03

a Group A: amiodarone group.

b Group P: placebo control group.

**Table 3.** Consumption of antiarrhythmic and vasoactive-inotropic agents.

Intravenous agents	Group A^a^ (N=27)	Group P^b^ (N=27)	*P* value
Esmolol (mg), median (IQR)	70 (65‐75)	130 (70‐150)	.20
Lidocaine (mg), median (IQR)	0 (0‐0)	0 (0‐0)	.36
VIS^c^ (T2^d^), mean (SD)	15.32 (3.66)	12.83 (4.19)	.02
VIS (T3^e^), mean (SD)	10.61 (3.99)	10.39 (4.10)	.84
TDA^f^, mean (SD)	32.20 (8.98)	27.54 (8.64)	.06

a Group A: amiodarone group.

b Group P: placebo control group.

c VIS: vasoactive-inotropic score.

d T2: fifteen minutes after weaning from cardiopulmonary bypass.

e T3: fifteen minutes before the end of surgery;

f TDA: total dose of vasoactive and inotropic agents.

### Hemodynamic Status During the Intraoperative Period

**Figure 2.**
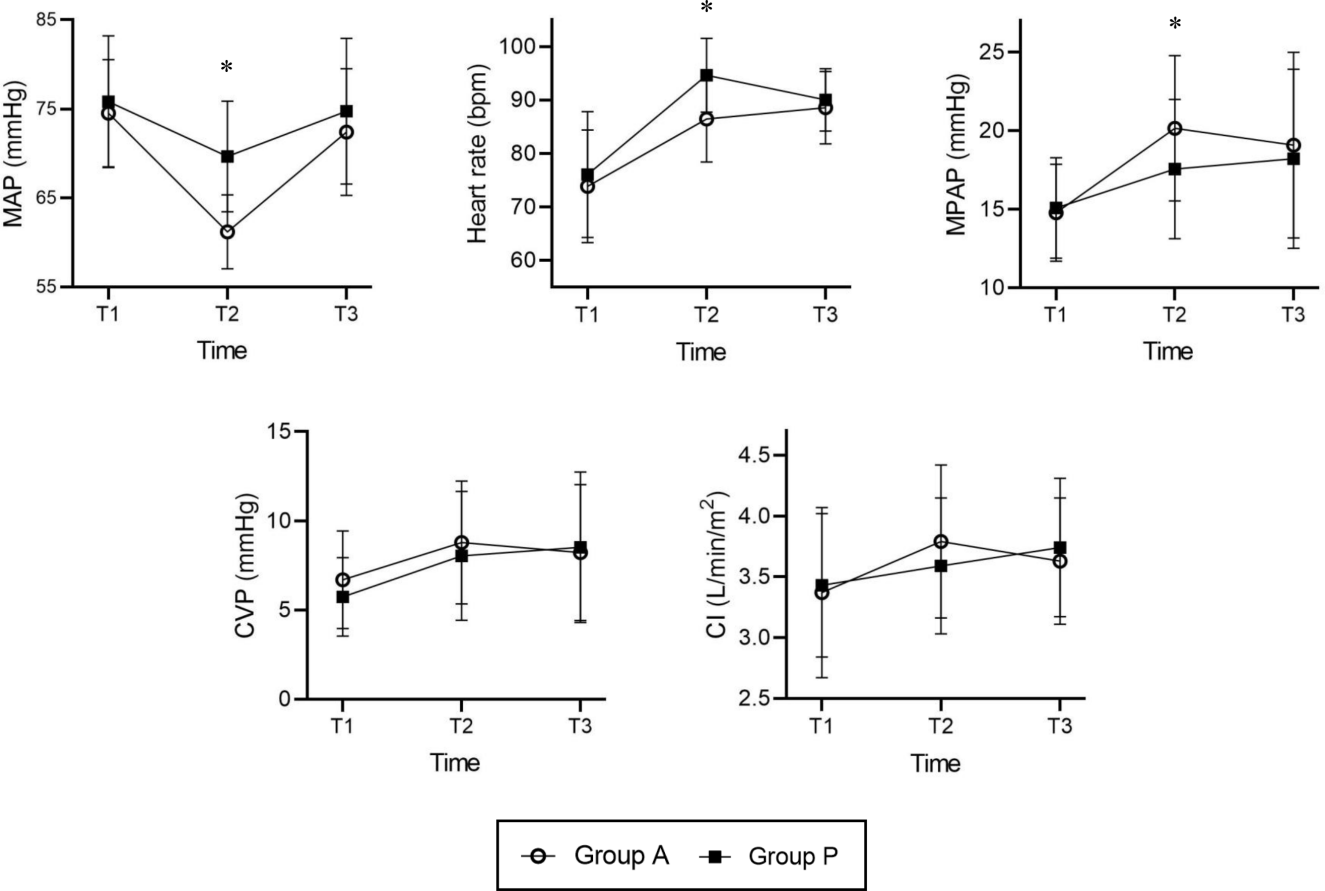
Changes in hemodynamic status during the intraoperative period. The global mean arterial pressure curve was lower in group A than in group P by repeated measures ANOVA. There were no significant differences in the global curves of heart rate, mean pulmonary arterial pressure, central venous pressure, and cardiac index between the 2 groups by repeated measures ANOVA. At T2 point, mean arterial pressure and heart rate were significantly higher in Group P while mean pulmonary arterial pressure was significantly higher in Group A. Group A: amiodarone group; Group P: placebo control group. CI: cardiac index; CVP: central venous pressure; MAP: mean arterial pressure; MPAP: pulmonary arterial pressure; T1: fifteen minutes after anesthetic induction; T2: fifteen minutes after weaning from cardiopulmonary bypass; T3: fifteen minutes before the end of surgery. *P<.05 was found between the two groups.

The hemodynamic status at various intraoperative times is shown in Figure 2. During the intraoperative period, repeated measures ANOVA indicated significant differences in MAP between the 2 groups (*F1*=5.161, *P*=.03). The global MAP was lower in group A than in group P (Figure 2). Compared with group P, MAP in group A was significantly lower at the time point T2 (mean 61.19 SD 4.16 vs mean 69.67 SD 6.23; *P*<.001). However, no significant differences were found in MAP at the time points T1 and T3 between the groups. During surgery, repeated measures ANOVA did not show significant differences in HR, MPAP, CVP, and CI between the groups (*F1,1,1,1*=3.098, 0.747, 0.271, and 0.001; and *P*=.08, .39, .61, and .97, respectively) (Figure 2). Compared with group P, HR (mean 86.44 SD 8.07 vs mean 94.63 SD 6.90; *P*<.001) was lower and MPAP (mean 20.15 SD 4.63 vs mean 17.56 SD 4.44; *P*=.04) was higher in group A at the time point T2. However, no significant differences were found in CVP and CI at the T2 time point. Furthermore, there were no significant differences in HR, MPAP, CVP, and CI at the time points T1 and T3 between the groups.

## Discussion

### Principal Findings

In this randomized trial, we primarily found that prophylactic intravenous use of amiodarone effectively reduced the incidence of VF in patients with LVH after ACC removal during open-heart surgery, while also decreasing the duration of VF, the frequency of defibrillation, and the energy required for defibrillation.

VF is a malignant arrhythmia that occurs after the release of the ACC in patients undergoing open-heart surgery. There are various factors that can contribute to VF after the removal of the ACC, which include surgical trauma, inadequate myocardial protection, insufficient intracardiac deairing or left ventricular venting, abnormal electrolyte and acid-base metabolism, delayed rewarming prior to ACC release, and myocardial reperfusion injury after ACC release. Among these factors, myocardial reperfusion injury is often considered the primary cause [16]. The prolonged duration of VF following ACC release can have several adverse effects on cardiomyocytes. Research findings indicated that VF led to alterations in the distribution of coronary artery blood supply, consequently causing subendocardial myocardial ischemia and subsequent cardiac dysfunction [17]. As the duration of VF extends, the resistance of subendocardial vessels progressively rises, exacerbating the subendocardial ischemia in cardiomyocytes. Furthermore, myocardial edema was observed to develop with the prolonged duration of VF [17]. During VF, there is an elevation in ventricular wall tension and venous pressure, necessitating a higher perfusion pressure to restore normal blood supply during CPB. The erratic and frequent myocardial contractions, along with elevated pressure in the cardiac cavity during VF, may lead to increased oxygen consumption by the myocardium. The described factors indeed result in an imbalance between oxygen supply and demand in the myocardium, leading to severe impairment of cardiomyocyte function.

In individuals with LVH, the cardiomyocytes enlarge in volume, yet the associated coronary arteries fail to grow in tandem, leading to a lack of subendocardial capillaries. Elevated end-diastolic pressure and enhanced systolic cardiac function contribute to an increase in myocardial oxygen consumption. These histological alterations result in an imbalance between myocardial oxygen supply and demand, causing subendocardial myocardial ischemia and reduced myocardial tolerance to ischemia-reperfusion injury [18]. Patients with LVH have been reported to be more likely to suffer from VF after removal of the ACC [5,6,13]. Therefore, this situation has created a need for prophylactic treatment.

Amiodarone, as a broad-spectrum antiarrhythmic agent, has several electrophysiological mechanisms to treat VF. The main antiarrhythmic effect of amiodarone occurs through the inhibition of IKr and IKs channels, leading to a homogeneous prolongation of myocardial repolarization [7]. In addition, it can reduce HR and slow atrioventricular nodal conduction by blocking calcium channels and β-receptors [19]. Furthermore, amiodarone extends refractoriness and decreases intracardiac conduction velocity by inhibiting the sodium channel [20]. During the period of ischemia-reperfusion, amiodarone has the ability to enhance the metabolic efficiency of cardiomyocytes and decrease the dispersion of repolarization across the cell membrane, which is a significant contributor to the development of ventricular arrhythmias [21].

Several clinical trials have investigated whether amiodarone could lower the occurrence of VF after ACC removal. Samantaray et al [11] reported that, compared with the placebo group, administering 150 mg of amiodarone 3 minutes before aortic opening during CABG surgery reduced the occurrence of VF after ACC release. In addition, Yilmaz et al [12] revealed that both 300 mg of amiodarone and 1.5 mg/kg of lidocaine reduced the occurrence of VF after ACC release compared with the placebo group. However, there were no significant differences in the reduction of VF between the amiodarone and lidocaine groups. On the contrary, in the clinical trial by Ayoub et al [22], it was found that the administration of amiodarone 2 minutes before aortic opening did not result in a reduction in the occurrence of VF after the release of the ACC during CABG surgery. On the other hand, lidocaine was effective in reducing the occurrence of VF in this study [22]. In addition, Mauermann et al [23] reported that both 300 mg of amiodarone and 1.5 mg/kg of lidocaine did not lead to a reduction in the occurrence of VF. Despite some conflicting results in the studies mentioned, there is a general indication that amiodarone has a therapeutic effect on VF after ACC removal.

In a documented case, a 66-year-old female patient underwent aortic valve replacement for severe aortic stenosis. The preoperative TTE screening indicated a thickness of up to 18 mm in the ventricular septum and left ventricular posterior wall. Following the release of the ACC and administration of lidocaine, refractory VF ensued, with multiple cardiac shocks showing no effectiveness. Finally, a dose of 150 mg of amiodarone was delivered through the aortic root, ultimately leading to the termination of VF with a single defibrillation [24]. Suzuki et al [5] also reported a similar case of using amiodarone to terminate rVF during cardiac surgery. In a clinical trial by Mita et al [13], patients with LVH undergoing aortic valve replacement were randomly divided into 2 groups. One group received a 150 mg bolus of amiodarone followed by a maintenance dose of 30 mg/h, while the other group received a 1 mg/kg bolus of lidocaine followed by a maintenance dose of 1 mg/kg/h after anesthetic induction. The occurrence of VF was markedly reduced in the amiodarone group compared with the lidocaine group (7/34, 20.6% vs 17/34, 50%; *P*=.02) [9]. Meanwhile, amiodarone has the potential to decrease the levels of interleukin-6 and tumor necrosis factor following the surgery.

In this study, our results showed that prophylactic intravenous amiodarone could lower the occurrence of VF in patients with LVH after the release of the ACC during open-heart surgery. Furthermore, among patients with VF, amiodarone reduced the number and total energy of internal cardiac shocks. In addition, the duration of VF could also be reduced. These results further proved the conclusions of the abovementioned studies. With the use of amiodarone, the recovery of cardiac contractions was not affected and there was no increase in the use of epicardial pacemakers. However, the duration of epicardial pacemakers was longer among patients with amiodarone, which may be correlated with the effect of amiodarone on slowing HR. In terms of hemodynamic effects, after weaning from CPB, HR and MAP decreased and MPAP increased, while the use of vasoactive and inotropic drugs was also higher in patients treated with amiodarone, which could be related to the pharmacological effect of amiodarone. However, CI and CVP were not affected and the hemodynamic status at the end of surgery was not significantly different between the groups. Regarding the consumption of vasoactive and inotropic drugs, although the dose of the drugs was higher among patients with amiodarone after weaning from CPB, the dose and total doses of vasoactive and inotropic agents at the end of surgery were not significantly different among the 2 groups.

### Limitations

Our study presents several limitations. First, this was a single-center randomized controlled clinical trial with a relatively small sample size, which limited the power of the statistical analysis. Second, lidocaine was not compared as a control group in the study, because it may be more effective than a placebo but less effective than amiodarone to reduce the incidence of VF according to previous studies [12,13], which could increase the sample size and the cost of the study. Third, this study only included patients with ASA I-III. Therefore, these findings cannot be generalized to patients with ASA IV, especially those with severe left ventricular dysfunction. Therefore, subsequent studies should further address the abovementioned issues.

### Conclusions

Preventive intravenous administration of amiodarone can reduce the incidence of VF in patients with LVH after ACC removal during open-heart surgery and decrease the duration of VF, defibrillation frequency, and defibrillation energy. It is safe and has little impact on intraoperative hemodynamic management. The findings may provide some insight for anesthesiologists in dealing with similar problems in clinical practice.

## Supplementary material

10.2196/64586 Checklist 1 SPIRIT (Standard Protocol Items: Recommendations for Interventional Trials) checklist.

10.2196/64586 Checklist 2 CONSORT-EHEALTH checklist (V 1.6.1).

## Abbreviations

ACC: aortic cross-clamp
ASA: American Society of Anesthesiology
CABG: coronary artery bypass grafting
CI: cardiac index
CONSORT: Consolidated Standards of Reporting Trials
CPB: cardiopulmonary bypass
CVP: central venous pressure
HR: heart rate
LVH: left ventricular hypertrophy
MAP: mean arterial pressure
MPAP: mean pulmonary arterial pressure
P_ET_CO_2_: end-tidal CO_2_
rVF: reperfusion ventricular fibrillation
TDA: total dose of vasoactive and inotropic agents
TTE: transthoracic echocardiography
VF: ventricular fibrillation
VIS: vasoactive-inotropic score
